# Optimization of Enzymatic Process for Vanillin Extraction Using Response Surface Methodology

**DOI:** 10.3390/molecules17088753

**Published:** 2012-07-25

**Authors:** Fenglin Gu, Fei Xu, Lehe Tan, Huasong Wu, Zhong Chu, Qinghuang Wang

**Affiliations:** 1 Spice and Beverage Research Institute, CATAS, Wanning, Hainan 571533, China; 2 National Center of Important Tropical Crops Engineering and Technology Research, Wanning, Hainan 571533, China; 3 Key Laboratory of Genetic Resources Utilization of Spice and Beverage Crops, Ministry of Agriculture, Wanning, Hainan 571533, China

**Keywords:** vanillin, extraction, response surface methodology, cellulose, extractionyield

## Abstract

Vanillin was extracted from vanilla beans using pretreatment with cellulase to produce enzymatic hydrolysis, and response surface methodology (RSM) was applied to optimize the processing parameters of this extraction. The effects of heating time, enzyme quantity and temperature on enzymatic extraction of vanillin were evaluated. Extraction yield (mg/g) was used as the response value. The results revealed that the increase in heating time and the increase in enzyme quantity (within certain ranges) were associated with an enhancement of extraction yield, and that the optimal conditions for vanillin extraction were: Heating time 6 h, temperature 60 °C and enzyme quantity 33.5 mL. Calculated from the final polynomial functions, the optimal response of vanillin extraction yield was 7.62 mg/g. The predicted results for optimal reaction conditions were in good agreement with experimental values.

## 1. Introduction

Vanilla (from *Vanilla planifolia* Andrews) is one of the most popular sweet spices in the World, and is now produced throughout tropical and subtropical areas. Due to its attractive sweet flavor, vanilla is widely used or consumed in a variety of products, including herbal cigarettes, alcoholic beverages, foodstuffs and cosmetics [1]. The major flavor constituent of vanilla extract is vanillin (4-hydroxy-3-methoxybenzaldehyde) [2]. Although vanillin extraction has been studied for over 60 years, enzymatic extraction of vanilla tincture has rarely been reported. 

Interest in the use of naturally-occurring flavors has increased dramatically in recent years, but since these natural flavorings belong to several different chemical classes, various extraction techniques must be used in order to obtain them [3]. Many botanical raw materials are rich in important fats, flavorings and coloring compounds, but these are often difficult to separate as they are sequestered by plant fiber, in particular cellulose which has a compact structure [4]. The use of enzymes to partially destroy the structure of the cell wall renders these intracellular compounds more readily available for separation and solvent extraction. Delgado-Vargas and Paredes-López [5] have reported that greater quantities of pigment could be extracted from fresh marigolds treated with cellulolytic enzymes than from untreated marigolds, with the highest yield of carotenoids obtained using econase-CEP, a commercially-available cellulase enzyme. Traditional methods of extraction use large quantities of organic solvent applied for prolonged periods of time, but nonetheless often yield a low extraction rate. A newer approach involves the use of enzymatic processing as a pretreatment step before extraction with an organic solvent; this method is simpler, uses less organic solvent and is more effective than the traditional method [6]. This technique thus shows genuine promise as an improved method for extracting components from plants.

Response surface methodology (RSM) is a collection of statistical and mathematical techniques useful for the improvement and optimization of complex processes. The main advantage of RSM is its ability to reduce the number of experimental trials needed to evaluate multiple parameters and their interactions, in order to provide sufficient information for statistically acceptable results [7,8]. It has been successfully demonstrated that this technique can be used for optimizing process variables [9,10].

Based on our preliminary experiments using this enzymatic technique, we have identified heating time, enzyme quantity and temperature to be key factors influencing extraction of vanillin. The overall objective of the present study was to investigate the effects of heating time, enzyme quantity and temperature on vanillin extraction, and to optimize the processing conditions using RSM to obtain high levels of vanillin.

## 2. Results and Discussion

### 2.1 Statistical Analysis

The experiments were carried out in a random order. The experimental plan was determined according to the quadratic regression rotation combination design. Values obtained from the extraction process are presented in Table 1, while characteristics of the model for extraction yield are shown in Table 2 and Table 3. ANOVA confirmed the adequacy of the statistical models, since their *p* values were less than 0.05, and they were statistically significant at the 95% confidence level. The models produced high determination coefficients (*R^2^* = 0.95) and low coefficients of variation (*CV* = 1.02%). These results indicate that the experiments carried out had good precision and reliability. The significance of each coefficient was determined by the t value and the *p* value, and these are listed in Table 2. The smaller the magnitude of the *p* value, the more significant is the corresponding coefficient. The fitted model equation was as follows (Equation 1):



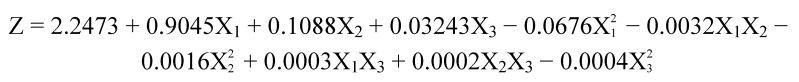
(1)


**Table 1:** Design program and experimental results of RSM.

Exp. number	Heating Time	Enzyme quantity	Temperature	Extraction yield
	/X_1_(h)	/X_2_ (mL)	/X_3_(°C)	/(mg/g)
1	7.78	43.31	77.8	6.96
2	7.78	43.31	42.16	6.94
3	7.78	23.69	77.8	7.17
4	7.78	23.69	42.16	7.25
5	4.22	43.31	77.8	7.08
6	4.22	43.31	42.16	7.04
7	4.22	23.69	77.8	7.01
8	4.22	23.69	42.16	7.19
9	9	33.5	60	7.11
10	3	33.5	60	7.01
11	6	50	60	7.13
12	6	17	60	7.31
13	6	33.5	90	7.27
14	6	33.5	30	7.41
15	6	33.5	60	7.63
16	6	33.5	60	7.53
17	6	33.5	60	7.59
18	6	33.5	60	7.65
19	6	33.5	60	7.57
20	6	33.5	60	7.67
*R^2^*				0.95
*CV* (%)				1.02

**Table 2:** Variance analysis of extraction yield.

Parameter	DF	Estimate	Standard Error	t Value	*p*-value	Parameter Estimate
					*Pr* > |t|	From coded data
Intercept	1	2.24726	0.668079	3.36	0.0072	7.609897
X_1_	1	0.904467	0.103066	8.78	<.0001	0.020774
X_2_	1	0.108797	0.018803	5.79	0.0002	−0.111128
X_3_	1	0.032427	0.010301	3.15	0.0104	−0.053873
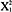	1	−0.067581	0.006165	−10.96	<0.0001	−0.608226
X_1_X_2_	1	−0.003150	0.001506	−2.09	0.063	−0.155912
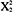	1	−0.001647	0.000204	−8.09	<0.0001	−0.448465
X_1_X_3_	1	0.000316	0.000829	0.38	0.7114	0.028413
X_2_X_3_	1	0.000229	0.00015	1.52	0.1592	0.113291
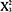	1	−0.000365	0.000061626	−5.92	0.0001	−0.328379

**Table 3:** Variance analysis of the second-order regression model on extraction yield.

Regression	DF	Sum of Squares	R-Square	*F* Value	*p*-value
					*Pr* > F
Linear	3	0.75522	0.0626	4.55	0.0295
Quadratic	3	1.036994	0.8601	62.44	<.0001
Cross product	3	0.037809	0.0314	2.28	0.142
Total Model	9	1.150324	0.9541	23.09	<.0001

### 2.2. Extraction Yield

As shown in Table 2, it was found that the extraction yield was positively related to the linear effects of heating time, enzyme quantity and temperature (*p* < 0.05), while the quadratic terms of heating time, enzyme quantity and temperature had a negative effect (*p* < 0.05). The interaction effects between heating time and enzyme quantity exerted a negative influence (Equation 1), but had no significant effects on extraction yield. The interaction effects between heating time and temperature, and between enzyme quantity and temperature, had positive influences (Equation 1), but no significant effects on extraction rate. 

**Figure 1:**
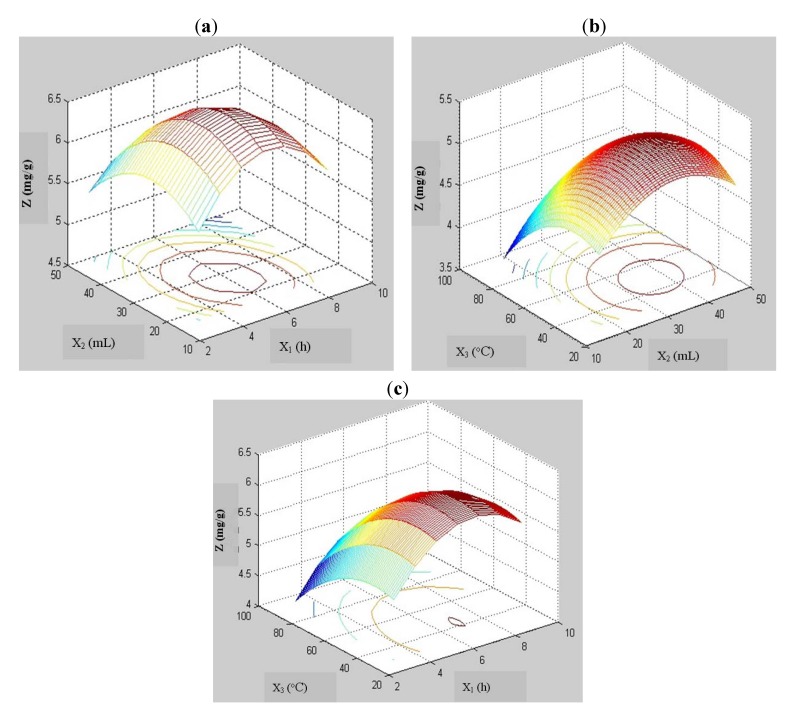
Three-dimensional figures of interactive effects of heating time (X_1_), enzyme quantity (X_2_) and temperature (X_3_) on extraction yield (Z). (**a**) Interactive effects of heating time (X_1_) and enzyme quantity (X_2_) on extraction yield (Z) at a fixed temperature; (**b**) interactive effects of enzyme quantity (X_2_) and temperature (X_3_) on extraction yield (Z) at a fixed heating time; (**c**) interactive effects of heating time (X_1_) and temperature (X_3_) on extraction yield (Z) at a fixed enzyme quantity.

Figure 1 depicts the response surface and contour plots of the effects of the three variables, namely heating time (X_1_), enzyme quantity (X_2_) and temperature (X_3_), on the extraction yield of vanillin. Figure 1a shows the dependence of extraction yield on heating time and enzyme quantity at a fixed temperature. It is clear that the extraction yield increased rapidly at the beginning of the experiment but then decreased slightly with increasing heating time; the same pattern was seen as the enzyme quantity was varied [11].

The variation of extraction yield with enzyme quantity and temperature, at a constant heating time, is presented in Figure 1b. It is evident that at a fixed heating time, the extraction yield increased rapidly with both enzyme quantity and temperature during the first stage of the experiment, but gradually decreased toward the end of the test. Furthermore, enzyme quantity was found to have a significant effect on the extraction yield of vanillin (*p* < 0.05). 

Figure 1c shows the effects of heating time and temperature on extraction yield at a fixed enzyme quantity. As heating time and temperature were varied, the extraction yield increased initially, but later decreased. 

These data indicate that the linear effects of heating time (*p* < 0.0001), enzyme quantity (*p* = 0.0002) and temperature (*p* = 0.0104), and the quadratic terms of heating time (*p* < 0.0001), enzyme quantity (*p* < 0.0001) and temperature (*p* = 0.0001), were dominant over the interaction terms. The interaction effects between heating time, temperature and enzyme quantity were not significant, although they appeared to slightly influence the extraction yield.

### 2.3. Optimization

The optimal processing parameters were determined in order to maximize the vanillin extraction yield. The extraction yield may be optimized from the figures (Figure 1a–c ). The zone of optimization depicts heating time to be in the range of 5.0–7.0 h, enzyme quantity to be in the range of 30–45 mL, and temperature to be in the range of 40–62 °C. The model describes the optimal conditions for extraction yield as: Heating time of 6 h; enzyme quantity of 33.5 mL; and temperature of 60 °C. The response (Z) calculated from the final polynomial function was an extraction yield of 7.62 mg/g. 

The suitability of the model equation for predicting the optimum response values was tested using the recommended optimum conditions. This set of conditions was determined to be optimum by a RSM optimization approach, which was also used to experimentally validate and predict the value of the responses using model equations. The experimental values were found to be in agreement with the predicted ones. The optimal extraction yield of vanillin in this experiment was less than 8.06 mg/g, as has been described previously [12]. This may be due to the different solvent, extraction time and pretreatment used; in the aforementioned study, the higher polarity of the solvent used may have facilitated the solubilization of vanillin (particularly glucovanillin), and use of heat pretreatment may also have improved vanillin extraction. In our study, pretreatment with enzyme dramatically reduced the time required for vanillin extraction from vanilla beans. The extraction time was far shorter than that of conventional methods, and RSM was shown to be effective for estimating the effects of processing variables [13].

## 3. Experimental

### 3.1 Materials

Vanilla beans (from cured vanilla pods of *Vanilla planifolia* Andrews) were obtained from the Spice and Beverage Research Institute (Hainan, China); the water and vanillin contents were 12.4% and 8.06 mg/g, respectively, determined using a method described previously [6]. Cellulase (15 U/mg) was purchased from Shanghai Bioscience and Technology Co., Ltd (Shanghai, China). Standard vanillin was purchased from Sigma Chemical Company (St. Louis, MO, USA). Methanol (HPLC grade) and other chemicals (analytical grade) were sourced from Shanghai Chemical Reagent Co., Ltd. (Shanghai, China).

### 3.2. Hydrolysis and Vanillin Extraction

Vanilla beans were ground in a centrifugal grinding mill (type ZM-1) fitted with a 1 mm stainless steel sieve (Retsch, Haan, Germany). 10 g vanilla powder was mixed with 50 mL acetic acid buffer (pH 4.8) and cellulase in a 250 mL Erlenmeyer flask. The flask was sealed and placed in a water bath, and the process of enzymatic hydrolysis was allowed to proceed with stirring using a magnetic stirrer (IKA Works Inc., Wilmington, NC, USA). Heating time, cellulase quantity and water bath temperature were varied to allow their effects on vanillin liberation to be studied. After hydrolysis, the reaction was stopped by 10 min immersion in boiling water, and the mixture was allowed to cool. 200 mL ethanol was then added, and the mixture stirred at 50 rpm for 12 h at 35 °C. Following this, the mixture was centrifuged with a Z36 HK Super Speed Centrifuge (Labnet, USA) at 10,000 × g for 30 min at 20 °C, to separate the solid and liquid phases. 1 mL of liquid phase was removed for vanillin quantification using HPLC. The yield of vanillin extraction was expressed using Equation 2.



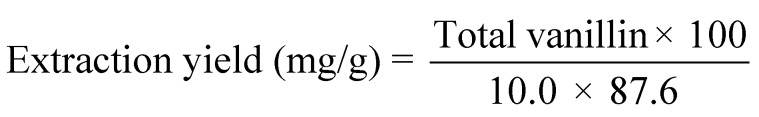
(2)


### 3.3. Determination of Vanillin by HPLC

Vanillin levels were quantified using a slight modification of methods described previously [4,6]. The HPLC system used was an Agilent 1260 series HPLC (Agilent Technologies, USA); separations were performed with a Zorbax Eclipse Plus C_18_ (4.6 mm × 100 mm, 3.5 μm) analytical column (Agilent Technologies) operating at 30 °C. A wavelength of 280 nm was considered the most appropriate for vanillin quantification, as maximal vanillin absorbance was found at this wavelength, and no interference was observed from other compounds present in the ethanol extract. A methanol/water (pH 4, acidified with acetic acid) mobile phase gradient was used, as follows: 0.8 mL/min of 60%/40% acidified water/methanol for 3 min; 1 mL/min of 65%/35% acidified water/methanol for 9 min; 0.8 mL/min of 60%/40% acidified water/methanol. The injection volume was 3.0 μL.

### 3.4. Experimental Design

According to our previous experimental findings, the most influential factors affecting extraction yield were heating time, enzyme quantity and temperature. In order to evaluate the effects and interactions of these three factors, RSM was used in designing the experiment. SAS software (version 9.0, SAS Institute Inc., USA) was used to generate the quadratic regression rotation combination design. The independent variables were heating time (X_1_), enzyme quantity (X_2_) and temperature (X_3_). Each independent variable had coded levels of −1.682, −1, 0, 1 and +1.682. This design was constructed based on a three-factor and a five-level Box–Wilson central composite design (CCD), leading to 20 sets of experiments, allowing each experimental response to be optimized. The experimental designs with regard to the coded (X) and actual heating times, enzyme quantities and temperature levels are shown in Table 4. The response (Z) was the extraction yield (mg/g). The response function Z was related to the coded variables (Xi, i = 1, 2, 3) by a second-degree polynomial equation using the method of least squares (Equation 3):



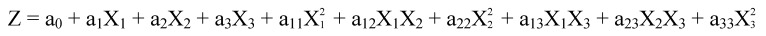
(3)


where Z is the response value calculated by the model; X_1_, X_2_ and X_3_ are coded variables, corresponding to heating time, enzyme quantity and temperature, respectively. a_1_, a_2_ and a_3_ are the linear; a_11_, a_22_ and a_33_ are the quadratic; and a_12_, a_13_ and a_23_ are the cross-product effects of the X_1_, X_2_ and X_3_ factors on the response.

Analysis of variance (ANOVA) was performed, ANOVA tables generated, and the effects and regression coefficients of individual linear, quadratic and interaction terms were determined. The statistical significance of the regression coefficients was determined using a T-test, and the applicability of the model was assessed using coefficient of determination (*R^2^*) and coefficient of variation *(CV*) values. The optimal processing conditions were obtained using graphical and numerical analyses, based on the criterion of desirability.

**Table 4:** Experimental design for the optimization of vanillin extraction.

Code levels	Variables		
	X_1_/Heating time (h)	X_2_/Enzyme quantity (mL)	X_3_/Temperature (°C)
r = +1.682	9	50	90
1	7.78	43.31	77.8
0	6	33.5	60
−1	4.22	23.69	42.2
−r = −1.682	3	17	30
△j	1.78	9.81	17.8

## 4. Conclusions

Response surface methodology is a useful tool to investigate the optimal conditions with regard to heating time, enzyme quantity and temperature, for extracting vanillin from vanilla beans. The coefficient of determination (*R^2^*) values for all parameters showed a good fit between the model and the experimental data, at the 95% confidence level. Analysis of the different conditions (heating time, enzyme quantity and temperature) for vanillin extraction revealed that heating time and enzyme quantity had a significant effect on extraction yield, while other variables showed an optimal zone for vanillin extraction. These results fitted well with the experimental data, and the models obtained can be used to optimize the values of the variables.
